# Bioactive Hydrogel Marbles

**DOI:** 10.1038/s41598-018-33192-6

**Published:** 2018-10-12

**Authors:** Álvaro J. Leite, Nuno M. Oliveira, Wenlong Song, João F. Mano

**Affiliations:** 1 3B’s Research Group – Biomaterials, Biodegradables and Biomimetics, University of Minho, Headquarters of the European Institute of Excellence of Tissue Engineering and Regenerative Medicine, Avepark – Parque de Ciência e Tecnologia, Zona Industrial da Gandra, 4805-017 Barco, Guimarães Portugal; 2 ICVS/3B’s - PT Government Associate Laboratory, Braga/Guimarães, Portugal; 3 The State Key Lab of Supramolecular Structures and Materials. Jilin University, Changchun, 130023 P.R. China; 4 Present Address: Department of Chemistry, CICECO, University of Aveiro, 3810-193 Aveiro, Portugal

## Abstract

Liquid marbles represented a significant advance in the manipulation of fluids as they used particle films to confine liquid drops, creating a robust and durable soft solid. We exploit this technology to engineering a bioactive hydrogel marble (BHM). Specifically, pristine bioactive glass nanoparticles were chemically tuned to produce biocompatible hydrophobic bioactive glass nanoparticles (H-BGNPs) that shielded a gelatin-based bead. The designed BHM shell promoted the growth of a bone-like apatite layer upon immersion in a physiological environment. The fabrication process allowed the efficient incorporation of drugs and cells into the engineered structure. The BHM provided a simultaneously controlled release of distinct encapsulated therapeutic model molecules. Moreover, the BHM sustained cell encapsulation in a 3D environment as demonstrated by an excellent *in vitro* stability and cytocompatibility. The engineered structures also showed potential to regulate a pre-osteoblastic cell line into osteogenic commitment. Overall, these hierarchical nanostructured and functional marbles revealed a high potential for future applications in bone tissue engineering.

## Introduction

The controllable manipulation of small volumes of liquids has attracted increasing interest due to the ongoing needs for miniaturized systems in biological applications^1^. In this regard, the principles behind natural repellent surfaces inspired the development of artificial nonstick liquid-solid interfaces, such as superhydrophobic surfaces^2^. Moreover, it has been demonstrated that the self-organization of hydrophobic powders on the air/liquid interface could encapsulate liquid droplets^3^. This phenomenon originates the so-called liquid marbles, which are defined as non-wetting soft objects comprising a liquid core shielded by micro or nanoscale particles. Therefore, the interaction with a solid substrate is converted to a solid/solid contact, mediated by the hydrophobic particles at the interface^4,5^. Thus, the hydrophobic shell prevents direct contact between the encapsulated liquid and surfaces outside the marble. Liquid marbles could then retain a stable shape while on the surface of a solid. Such systems made possible straightforward strategies for an easy handling of liquids. The unique properties of liquid marbles have made them suitable in many fields, such as materials science, biochemistry, and in biomedicine^6,7^. They became successfully applied in the production of 3D microreactors^8^, cell spheroids^9^, *in vitro* tumors models^10^, high-throughput screening^11^, microorganisms cultures^12^, rapid blood typing^13^, chemical sensors, micropumps, pollution and gas detection^14^, and even in cosmetic products^15^.

However, the hydrophobic shell is only seen to confine and protect the liquid core from the external environment, without further biological interaction and bio-functionality. Pioneering works have employed distinct coatings to behave as conventional actuators through *ex-situ* stimuli (such as pH, light, temperature, and magnetism) that merely alter the mobility of liquid marbles or just switch off the confinement of the liquid core^16^. Moreover, the involved stimuli may detriment some bio-functions and thus be inapplicable for the handling of cargos containing bio-sensitive agents^17,18^. Hence, it is compulsory to develop multifunctional shells that can be naturally modulated without harsh stimuli. Despite useful in many applications, the liquid core hinders the necessary matrix to sustain cell growth and proliferation when cell delivery therapies are considered. Moreover, there is still a need for a technology that enables cells to grow in three dimensions in their native state without the restriction of common scaffolds, thus mimicking their niche and closing the gap with the cells behavior in the *in vivo* scenario^19,20^.

Bearing this in mind, we envisage a bio-functional shell formed by bioactive glass nanoparticles (BGNPs) for bone regeneration. It has been shown that liquid marbles created with nanoparticles appeared more mechanically robust than those made from microparticles due to a more uniform shielding^21^. Additionally, bioactive glasses could form a calcium phosphate apatite that integrates to *in vivo* bone tissue, accelerating osteointegration^22,23^. Also, the ionic release from nanosized bioactive glasses could stimulate gene expression and promote osteoinduction^24^.

The extracellular matrix of hard tissues is typically formed by organic and inorganic phases. In this sense, polymer-based nanocomposites containing BGNPs are an attractive approach to better mimic the native bone structure^25^. Natural-derived hydrogels are commonly used in biomedical applications due to their similarity with macromolecules present in the biological environment, matching the mammalian tissue niche morphologically and physiologically^26,27^. Hydrogels can be processed using cell-friendly encapsulation parameters such as mild temperature and shear forces (attenuating the mechanical stress on encapsulated cells), while allowing the bidirectional diffusion of oxygen, nutrients, and metabolic waste.

Therefore, we propose a strategy to produce hydrogel microspheres with a bioactive shell by exploiting the liquid marble methodology and the self-cleaning effect of natural repellent surfaces. The rationale resides on the possibility of fast producing well-designed bioactive marbles where cells and drugs can be incorporated. In this regard, BGNPs were chemically tuned to obtain hydrophobic nanoparticles (H-BGNPs). Gelatin methacrylate was used to produce droplets that rolled over a superhydrophobic substrate, collecting H-BGNPs that stacked at the droplet surface. Thus, the polymeric beads became shielded originating bioactive hydrogel marbles (BHM). The whole BHM manufacturing process was designed under mild conditions, where no complex instruments were needed.

## Results

### Production and characterization of the hydrophobic bioactive glass nanoparticles (H-BGNPs)

**Figure 1:**
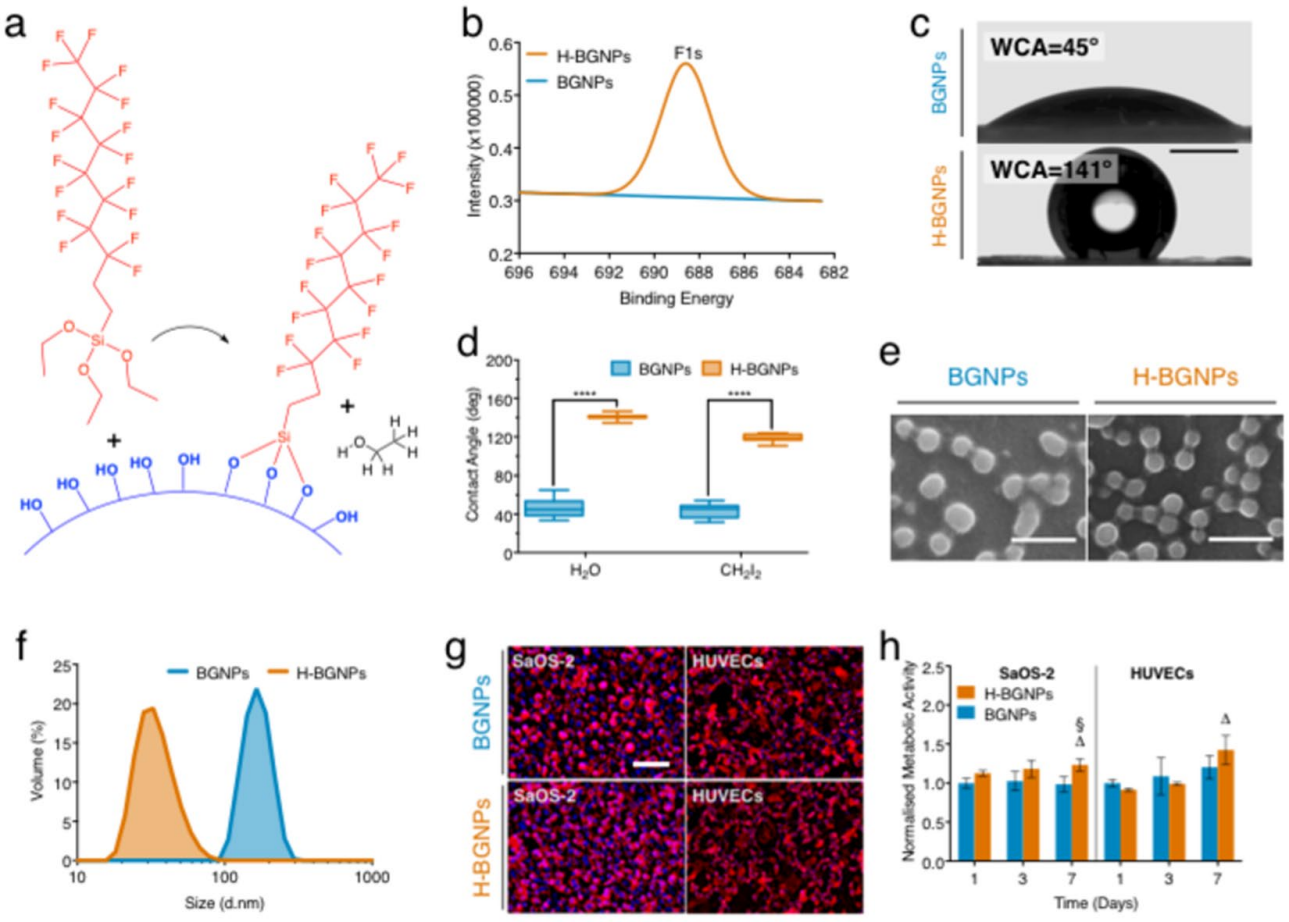
Production and characterization of the hydrophobic bioactive glass nanoparticles (H-BGNPs). (**a**) Illustration of the synthesis of the novel H-BGNPs through nature-inspired chemistry: the theoretical grafting of PFDTS molecules on the BGNPs surface. (**b**) X-ray photoelectron spectroscopy (XPS) analysis of the nanoparticles before and after grafting by PFDTS (H-BGNPs), which confirmed the chemical functionalization. An EDS spectra are also present in Fig. S1. (**c**) Water contact angle (WCA) of the BGNPs and H-BGNPs. Scale bar = 1 mm. The BGNPs could be wetted by water due to the hydroxyl groups on its surface. The fluorosilanization of the BGNPs renders the initial hydrophilic nanoparticles into hydrophobic bioactive glass nanoparticles. (**c**) The contact angle of BGNPs and H-BGNPs. (**e**) SEM micrographs of H-BGNPs and BGNPs. Scale bar = 500 nm. (**f**) Evaluation of the size distribution by dynamic light scattering (DLS). (**g**) Representative photographs of SaOS-2 and HUVECs at 7days of cell culture. Scale bar = 200 µm. The full comparison with non-modified BGNPs is presented in Fig. S3. (**h**) AlamarBlue assay results, presenting the metabolic activity of both cell types for 1, 3, and 7 days of cell culture.

Pristine bioactive glass nanoparticles (BGNPs, SiO_2_:CaO:P_2_O_5_ (mol%) = 55:40:5) are intrinsically hydrophilic. In this regard, fluorine-based molecules have been used to lower the surface energy of silica-based compounds^28^. Therefore, a fluorosilane was grafted on the nanoparticles by taking advantage of the surface hydroxyl groups (Fig. 1a)^29^. Consequently, and due to the reactive and polar nature of the fluorine groups, hydrophobic bioactive glass nanoparticles (H-BGNPs) were produced. The chemical analysis revealed that the functionalized nanoparticles comprised fluorine in its composition (Fig. 1b), confirming that the grafting process was accomplished. Moreover, the functionalization led to a decrease in oxygen and silicon content and an increase of the carbon content (Table S1), validating the grafting hypothesis. However, the experimental atom ratio of F to Si (AR_F/Si_) was 1.14 ± 0.54, which unmatched the theoretical value of PFDTS (AR_F/Si_ of 17.0). As fluorine was exclusively originated from the PFDTS molecule, this discrepancy indicates the incomplete shielding of the nanoparticles. Such finding would be necessary for the following studies as it will allow the interaction of the nanoparticles with physiological like solutions. Due to the changes in BGNPs surface chemistry by fluorosilanization, the initial hydrophilic nanoparticle became hydrophobic with a water contact angle of 141° ± 1.3° (Fig. 1c,d). Thus, the nanoparticles’ surface free energy decreased (Table S2), agreeing with previous studies^30^. The desirable hydrophobicity finds parallelism in nature as it could be ascribed to these aspects: (1) H-BGNPs were organized to create a rough surface mimicking the hierarchal structure of lotus leaf; (2) the grafted PFDTS served as the chemical structure to mimic botanical wax of lotus leaf to gain low surface free energy^31^. Having achieved a successful functionalization and the desired hydrophobicity, the morphology of the nanoparticles was investigated. The H-BGNPs exhibited a spherical shape, and no significant differences were found in their morphology compared to pristine nanoparticles (Figs 1e and S2). Furthermore, the H-BGNPs showed a variable size, ranging from 8 to 100 nm, which is slightly smaller compared with pristine nanoparticles (40-200 nm, Fig. 1f). This evidence might be explained by the fact that the fluorosilanization prevented the aggregation of nanoparticles.

To move from the bench to the clinics, biomaterials must be non-toxic to cells, particularly to the ones related with new bone formation. Simultaneously, the vascularization process should occur, to supply oxygen, nutrients, and growth factors, vital to cell survival, integration, and successful regeneration. This interrelation is described as the angiogenic-osteogenic coupling^32^. Bearing this in mind, the biological response of the bioactive glass nanoparticles was investigated employing two distinct cell types, SaOS-2 and HUVECs (Fig. 1g). A clear difference in the proliferation and spreading is present on day 1 and 3 (Fig. S3). We hypothesize that this difference is ascribed to the hydrophobic character of the nanoparticles in early stages as it could reduce protein/cell adsorption^33^. As the culture time progresses this effect vanishes due to either ionic dissolution of the nanoparticles or adhesion of proteins. At 7 days, the levels are equalized. An increase in the metabolic activity throughout the culture period was also detected for both cell types (Fig. 1h). Moreover, no significant adverse effect on the natural metabolic activity was noticed until 7 days of culture, for either SaOS-2 or HUVECs. Therefore, H-BGNPs were employed for the further fabrication of marbles because of their tailored hydrophobicity and proved biocompatibility.

### Production of the hydrogel marble

**Figure 2:**
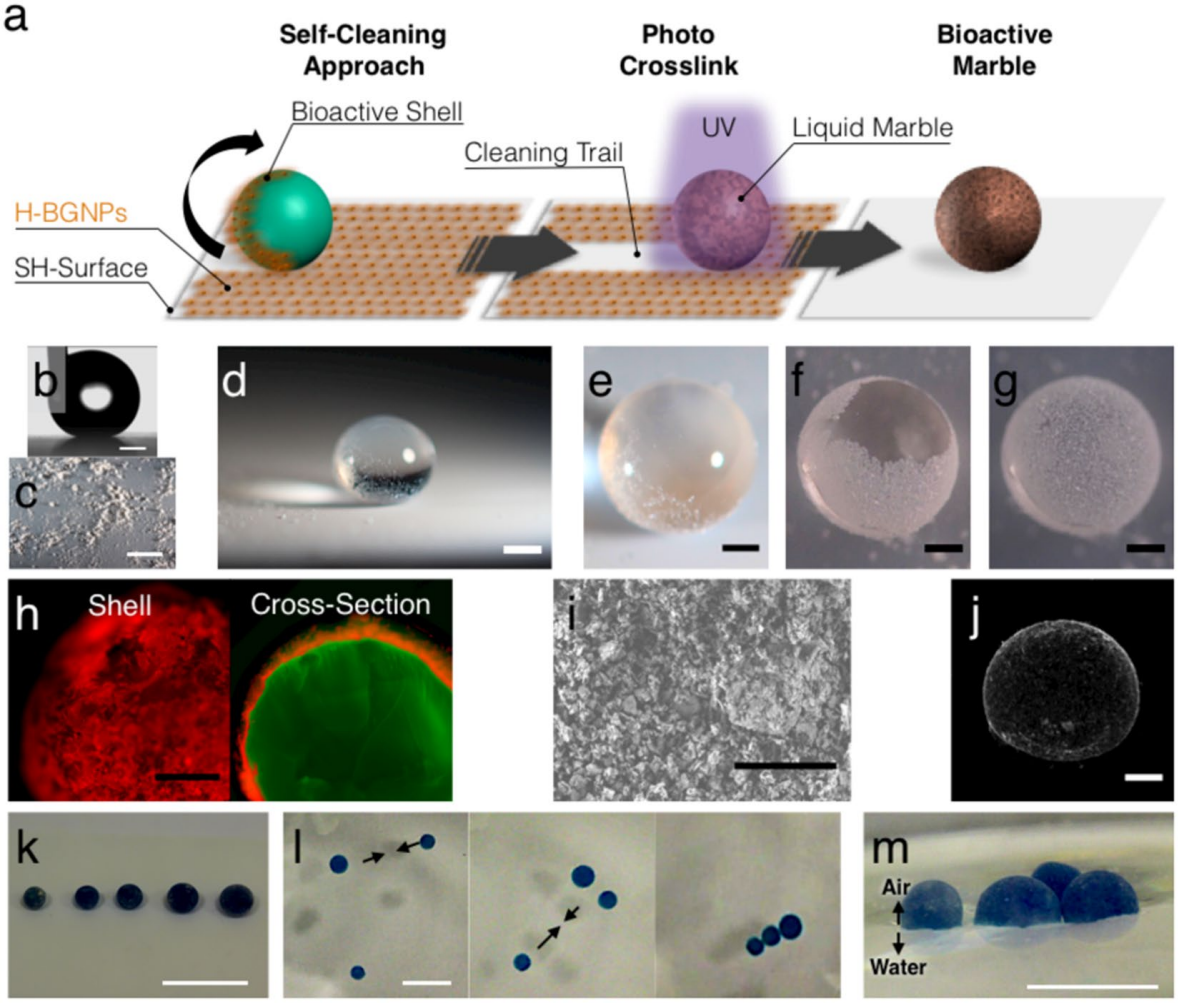
Fabrication of the hydrogel marble. (**a**) Schematic drawing of the synthesis procedure of the bioactive hydrogel marble by a biomimetic approach based on the self-cleaning of the lotus leaf. (**b**) Profile of a water drop in contact with the superhydrophobic surface. Water contact angle of 162 ± 1.3°. Scale bar = 500 µm. (**c**) Superhydrophobic surface covered with H-BGNPs. The observed clusters could be attributed to powder electrostatic effects. Scale bar = 1 cm. (**d**) Polymeric drop on the superhydrophobic surface. Scale bar = 500 µm. (**e**) Detail of the polymeric drop rolling on the superhydrophobic surface. Scale bar = 500 µm. (**f**) Formation of the bioactive shell. Scale bar = 500 µm. (**g**) Bioactive marble: polymeric sphere covered with H-BGNPs. Scale bar = 500 µm. (**h**) Fluorescent micrographs detail of the shell and cross-section of the bioactive marble. Before the fabrication of the bioactive marble, the gelatin and the H-BGNPs were first stained with fluorescein (green) and rhodamine (red), respectively. Scale bar = 500 µm. (**i**) Representative SEM micrographs of the H-BGNPs at the surface of the BHM. Scale Bar = 5 µm. (**i**) μCT 3D reconstruction images of the BHM. (**k**) Photographs showing BHM (dyed in blue) on top of a superhydrophobic surface. The dispensed volumes were 1.5, 2, 4, 8, and 16 μL. The approximate diameters of the spheres after crosslinking were 1, 1.2, 2.5, 4; 5; 6 mm, respectively. Scale bar = 1 cm. (**l**) BHM floating at the surface of the water. Scale bar = 0.5 cm. (**m**) Representative image of the floating performance of the BHM cluster that could be manipulated and self-assemble on the surface of the water. Scale bar = 1 cm.

Inspired by natural self-cleaning strategies (Fig. S4a), we developed a manufacturing method to produce microspheres with a nanostructured shell (Fig. 2a). First, a biomimetic procedure was used to prepare superhydrophobic platforms (SH-surface, Fig. 2b), in where small volumes of a polymeric aqueous solution could acquire a droplet configuration. Herein, gelatin methacrylate was chosen for the proof-of-concept as it is a photocrosslinkable polymer with proven biocompatibility^34^. On their path to roll over the SH-surface covered with H-BGNPs (Fig. 2c), the polymeric droplets collected the nanoparticles (Fig. 2d,e and Video S1). The H-BGNPs, absorbed at the air-liquid interface, aggregate due to capillary forces created by the deformation of the liquid surface (“Cheerios effect”)^35,36^. As a result, a nanostructured film was encapsulating the droplet (Fig. 2f). This created a semitransparent liquid marble (Fig. 2g), which is coherent with studies using nanoparticulate coatings^21^. The nanostructured shell prevents a direct contact between the liquid core and external surfaces, creating a robust soft solid. Moreover, the obtained marbles retained a stable shape and could act as microcarriers by moving randomly without leakages. Then, the polymeric liquid core was cross-linked by UV light, forming a bioactive hydrogel marble (BHM). The adhesion of H-BGNPs only at the outer surface of the hydrogel microsphere and the consequent shielding was microscopically confirmed (Fig. 2h). Notably, due to the capillary forces, the H-BGNPs compacted into a tight layer (Fig. 2i), a phenomenon well observed with the classical “liquid marbles”^3^. Moreover, the BHM presented a quasi-spherical shape (Fig. 2j), because of their previous deformation under the gravity effect^37^. The quantity of the polymeric solution dispensed using a micropipette could define the volume of the produced BHM (Fig. 2k). However, a high volume resolution may be achieved using other dispensing systems, such as spraying^38^.

The contact of the bioactive marble with water did not lead to the dissociation of bonded H-BGNPs from BHM surface, but only those loosely attached (excess). Additionally, the engineered BHM could float with remarkable mobility and self-assemble on the surface of the water (Fig. 2l). The driving force for this capability arises from the capillary interactions between the floating BHM^39^. When the convex menisci are within the range of the capillary length of a liquid, the marbles become prone to attract each other^40^. This principle is also confirmed as the BHM with higher volume (higher meniscus deformation) join faster together (Video S2). Moreover, the self-assembled BHM continues to exhibit a water-repelling capacity, as demonstrated by the convex meniscus formed by the surrounding water when it was placed at a water-air interface (Fig. 2m). The controlled manipulation of small volumes is important to construct miniature systems for chemical and biological applications. There have been published several reports on actuating liquid marbles with an external electric and magnetic field or acoustic action^37^. However, this self-assembly capacity potentiates the simple scale-up for microstructured hydrogels to fabricate large pieces of interconnected tissues^41,42^.

Such floating capacity of the BHM might be explained by an intermediate wetting condition of the classical Wenzel and Cassie-Baxter states^43^. In where the water could infiltrate into the microstructures formed by the H-BGNPs while keeping an amount of air sealed between the cavities. This explanation follows other works^7^ and premises a fluid exchange between the hydrogel core and the surrounding aqueous environment enabled by capillary and/or osmotic forces^44^. Therefore, the eventual decreasing on floating ability of the BHM over time could be attributed to the proved swelling of gelatin methacrylate. The initial packed layer of H-BGNPs became sparse when the volume of the hydrogel core expanded, resulting in the decrease of the collective water repellency and an increase resemblance with pristine hydrogels, leading to the sinking of the BHM.

### The bioactive behavior of the engineered marbles

**Figure 3:**
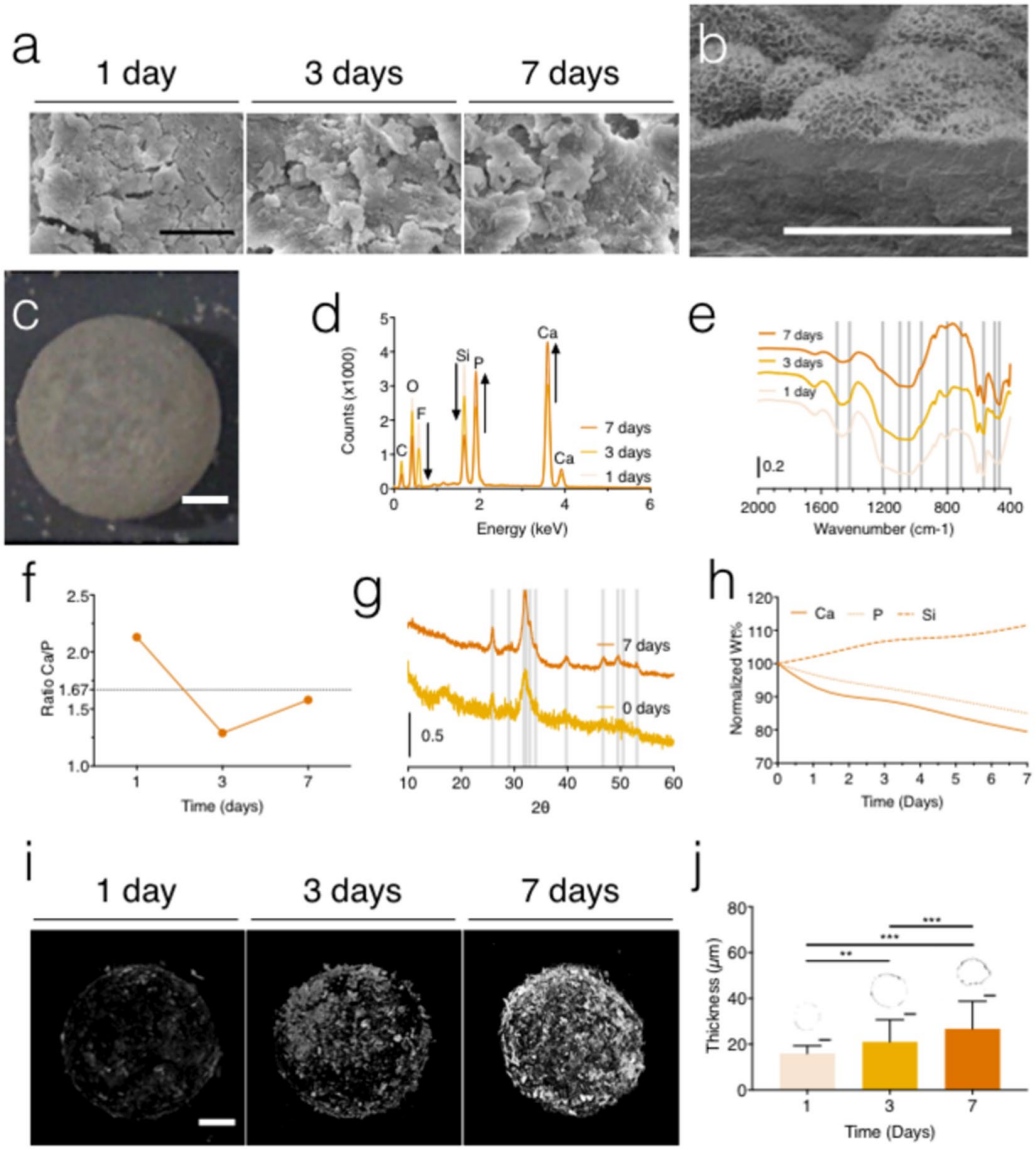
The bioactive behavior of the produced marbles. (**a**) Representative SEM micrographs of the bioactive marbles soaked in SBF for 1, 3, and 7 days. Scale bar = 5 µm. (**b**) Cross-section SEM micrograph of the hydrogel marbles soaked in SBF after 7 days, exhibiting the aggregates of nanometric needle-like crystals that characterized cauliflower morphology of hydroxyapatite. Scale bar = 5 µm. (**c**) Detail of the hydrogel marbles soaked in SBF after 7 days. Scale bar = 500 µm. (**d**) Identification of chemical elements performed by EDS following testing of BHM in SBF during 1, 3, and 7 days. (**e**) FTIR spectra of the apatite development on the surface of the bioactive marble. The chemical groups and the bandwidths used for identification are specified in Table S3. (**f**) Ca/P ratio during 1, 3, and 7 days of immersion in SBF. (**g**) XRD spectra of the BHM obtained before and after the immersion in SBF (0 and 7 days). The main characteristic hydroxyapatite peaks are shown at 2θ = 25.9°, 29°, 31.8°, 32.2°, 32.9°, 34°, 39.8°, 46.7°, 49.5°, 50.5° and 53.1° ^68^. (**h**) ICP study about the variation of Ca, Si and P in the SBF along of 7 days. (**i**) μCT 3D reconstruction images of the bioactive marbles after 1, 3, and 7 days of immersion in SBF. Scale bar = 500 µm. (**j**) The thickness of the bioactive marbles based on the μCT during 1, 3, and 7 days of immersion in SBF and the representative cross-sections images. Scale bar = 500 µm.

Biomineralization studies assessed the osteoconductive potential of the designed BHM and their capacity for tissue engineering applications. Therefore, the ability of BHM to form a surface apatite layer was tested *in vitro* by immersion in SBF. This is a well-established test to predict the interaction with the surrounding biological tissue in the implanting scenario^45^. After 1 day of immersion in SBF, the H-BGNPs already induced the precipitation of mineral agglomerates at the surface of the BHM that densified with time (Fig. 3a). This gradual growth of a mineral layer on soaked BHM indicates the formation of an apatite precipitate. Indeed, after 7 days, the apatite layer that enclosed the entire outward of the marble resembled the characteristic cauliflower morphology of hydroxyapatite (Fig. 3b). Consequently, the initial translucent BHM became opaque (Fig. 3c), which imitates nature structures (Fig. S4b). Chemical analysis monitored the mineralization on the surface of the BHM. The surface of the marbles demonstrated a steady growth of relative amounts of P and Ca, with a gradual reduction in the Si and F relative levels, along with the 7 days of immersion (Fig. 3d). Besides showing the characteristic vibrations of the groups on the H-BGNPs structure, the BHM coating also exhibited bands attributed to the mineralization process, such as the stretching vibration of phosphate groups around 600 cm^−1^ that, after 7 days, changes for two bands (607 and 570 cm^−1^, Fig. 3e). Indeed, the formation of the P-O pair could suggest the presence of a carbonated bone-like apatite^46^. The observed chemical evolution is typical of the surface of bioactive glasses in physiological fluids due to two concurrent processes: their ionic dissolution and the deposition of calcium phosphate ceramics^47^. Therefore, the development of depots with time is associated with the longer period available for the precipitation of apatite. These outcomes validate the settlement and densification of the apatite layer, which is supported by the SEM micrographs and concordant with former reports^48^. The degree of calcification could be evaluated by the Ca/P atomic ratio. The Ca/P ratio approximated to the stoichiometric theoretical value of hydroxyapatite (1.67): 1.29 after soaking during 3 days and 1.58 after only 7 days (Fig. 3f). This earlier apatite development was predictable, because of the augmented ion dissolution kinetics potentiated by the nanosize dimension of H-BGNPs^49^. Moreover, the apatite formed after 7 days resembled the characteristic semi-crystalline hydroxyapatite diffractogram (Fig. 3g). Agreeing with previous studies, this observation shows the development of carbonated bone-like hydroxyapatite^50,51^. The depletion of relevant ions from the SBF solution was analyzed to comprehend the dissolution rates of the H-BGNPs. With time, the concentrations of P and Ca declined due to their utilization in the growth of the bone-like apatite (Fig. 3h). Simultaneously, in the SBF solution, the concentrations of Si grew until reaching stable values. In this sense, as H-BGNPs could release soluble SiO_2_ into the SBF solution, these results might be assigned to the condensation and re-polymerization of the SiO_2_-rich layer on nanoparticles exterior. The dissolution kinetics of the H-BGNPs reflected the EDS results and attested the formation of a bone-like apatite coating around BHM^52^. The 3D density analysis demonstrated that the mineralization occurred only at the surface of the BHM coinciding with the localization of the H-BGNPs (Fig. 3i and Video S3). After 7 days, the bone-like apatite depots could cover the entire surface of the BHM. Moreover, the thickness of the marble coating increased over the 7-days (Fig. 3j), attesting the densification of the bone-like apatite layer. Therefore, the arrangement of the nanoparticles at the surface of the polymeric core provided an exceptional tailoring of the osteoconductive capacity of the newly developed BHM. Overall, and besides affecting the hydrophilicity, the coating of H-BGNPs can confer osteoconductivity to BHM once it induced a bone-like apatite layer on marble’s surface in a physiological-like environment.

### Biological functional performance of the BHM

**Figure 4:**
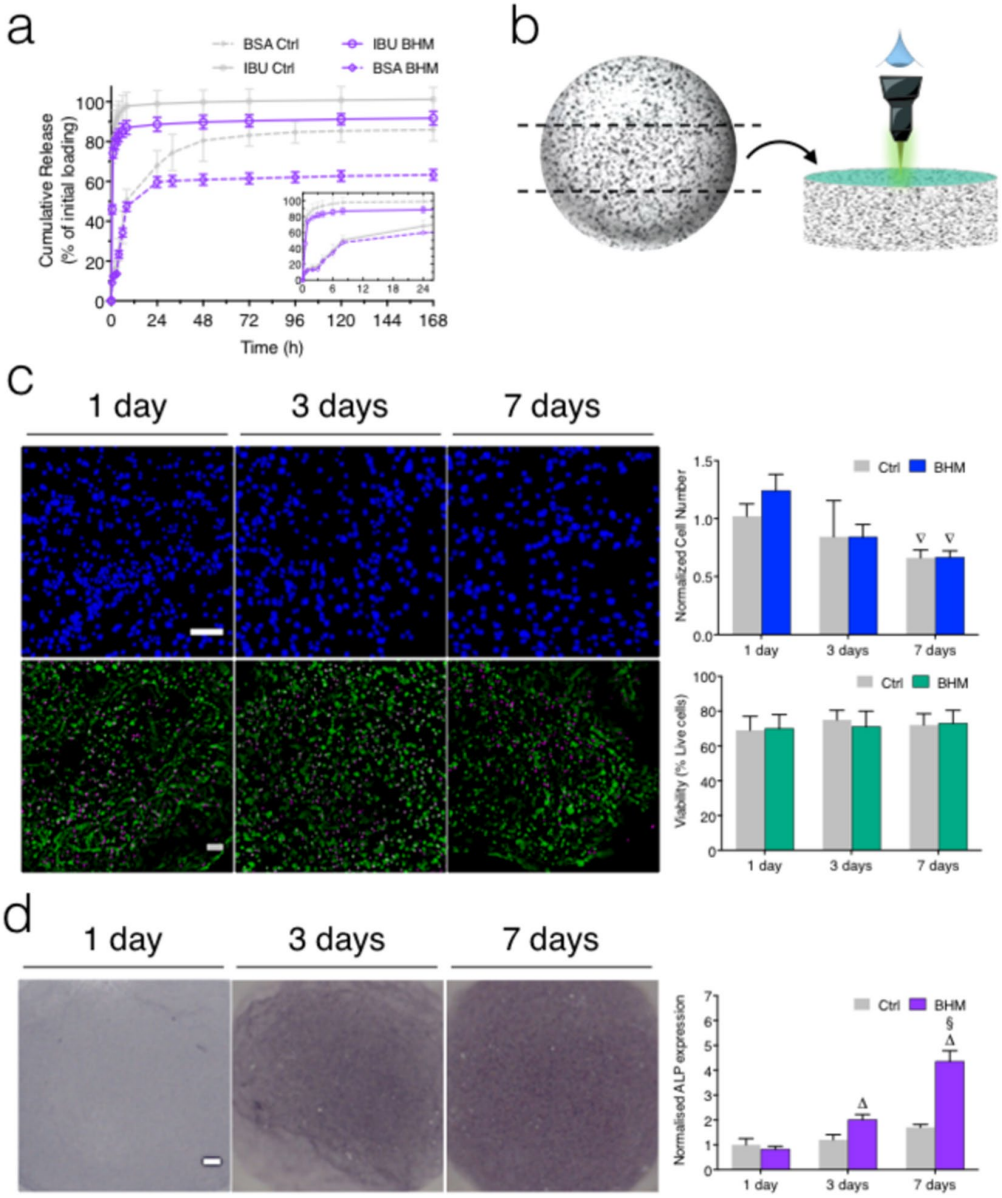
Biological functional performance of the produced bioactive hydrogel marbles and the comparison with non-coated hydrogel spheres (Ctrl). (**a**) BSA and Ibuprofen accumulative release profiles from the constructs. The inset graphic amplifies the release profiles in the first 24 hours. (**b**) Slice of the BHM for core imaging purposes to eliminate the shell artifacts. (**c**) DAPI (on top) and live-dead (at the bottom) fluorescent microscopy images (blue represents nuclei, green represents live cells, and magenta represents dead cells) of MC3T3-E1 cells. Scale bar = 100 µm. The corresponding quantification by image analysis is also shown. The images of non-coated hydrogels (Ctrl) are presented in Fig. S7. (**d**) Evaluation of the early osteogenic commitment with representative images of ALP staining during the cell culture time and the respective image analysis quantification. Scale bar = 100 µm. When applicable the results are presented as the arithmetic mean ± standard deviation. The images of non-coated hydrogels (Ctrl) are presented in Fig. S8.

Regenerative medicine requires systems that can load and offer a controlled release of therapeutic agents. Moreover, the synergistic action of combined drug therapies could lead to superior healing efficacy and reduce drug resistance. In this regard, the potential of the BHM for loading and *in situ* release of drugs was explored. The asset of the self-cleaning setup is that the marbles were processed with no extraneous liquid medium. Thus, the drug loaded into the polymeric precursor remained constant in the final BHM^53^. Ibuprofen (IBU) and albumin (BSA) were selected as model drugs with distinct molecular weights (Fig. 4a). The IBU released immediately in the first 12 h (burst effect), reaching a plateau after just 24 h and consistent with Fickian diffusion (Fig. S5). However, a lower amount of IBU was released when compared with non-coated hydrogel spheres (P < 0.01). This observation could be explained by the interaction between IBU and H-BGNPs. Works have stated that the IBU might form hydrogen-bonded complexes with the silanol groups on silica surfaces and with the growing hydroxyapatite layer on the BHM shell^54,55^. BSA had a slower release profile than IBU (P < 0.001) with a complete release around 60% that appeared to be governed by diffusion and swelling (Fig. S5). We consider that due to the hydrogel network the release of the BSA was more impaired. Such kinetics follow the typical profiles of low and high molecular weight agents from hydrogel systems, mediated by an unimpeded diffusion across the hydrated matrix^56^. More interestingly, when compared with non-coated hydrogel spheres (P < 0.001), the release of BSA ceased between 1 and 3 days, indicating that the bioactive shell induced a “cut-off”. Therefore, the BHM could behave as a functional system in where the mesh of the hydrogel core controls the rate of release while the shell endeavors a time-dependent selectiveness. Additionally, the polymer concentration, initiator concentration and UV exposure time are the major parameters that allow tuning of the physical properties of the hydrogel core and, consequently, that allow creating the required release profile for specific demands by tailoring of diffusion and swelling behavior^57^. This ability of dual-release could be useful in many therapies that need a differentiated release of pharmacological agents, such as anti-inflammatory, antibiotics or molecules to control cell behavior of encapsulated cells. In this regard, MC3T3-E1 cells were used as an osteoprecursor model, which are known to be recruited from circulation to bone defect sites^58^. The BHM were then sliced for core imaging purposes, eliminating the shell artifact (Fig. 4b). The BHM presented a significant variation in cell number after 7 days that was attributed to the artifact of the swelling of the hydrogel core (Fig. 4c). Cells were uniformly distributed within the hydrogel core (Fig. S6). High cell viability rates, ranging from 70-95%, were observed (Fig. 4c). No differences were found between the BHM and pristine hydrogels, proving that the shell of H-BGNPs did not influence cell fate. Such high viability of the encapsulated cells indicated a favorable 3D microenvironment inside the hydrogel core, proving that the nutrient exchange between the BHM and the surrounding cell culture medium was sufficient. Therefore, the hydrogel core could provide a 3D environment that supports cell-matrix interaction and imitates the native tissue^20^. Moreover, MC3T3-E1 cells are reported to exhibit behavior analogous to *in vivo* bone development. We analyzed the alkaline phosphatase (ALP) activity of the encapsulated cells, as it is a well-accepted early marker for the osteogenic differentiation^59^. ALP is expressed during the post-proliferative period and is a marker of early differentiation and extracellular matrix mineralization^60^. The BHM promoted an increased ALP expression after 3 and 7 days of cell culture (Fig. 4d). Also, after 7 days of culture, the normalized ALP values were higher than the control. Considering that the BHM were cultured in completely basal conditions, this behavior became comparable to the one observed in cell cultures supplemented with osteogenic inducers^61^. Interestingly, correlation analysis showed that ALP expression depended on the BHM osteoconductive behavior (particularly on the effect of Ca, P, and Si, Tables S4 and S5). Possibly, the nanostructured shell, provided by the H-BHNPs, might translate into a rate of ion dissolution that provided the necessary concentrations levels of Si and Ca for the osteogenesis of the encapsulated cells. This effect is consistent with studies on ionic release products from bioactive bioglasses^62^ and on reports on the effect of ions on osteoblasts^63^. A comprehensive scheme regarding the performance of the design BHM is present in Fig. S9. Further, by combining the cell encapsulation capacity with the self-assembly capability of the BHM, biomimetic 3D multi-tissue constructs could be produced. Therefore, the designed BHM could be a solution when scaffold-free approaches are sought for musculoskeletal tissue regeneration (Fig. S10).

## Discussion

Inspired by the self-cleaning of the lotus leaf, we have developed an innovative core-shell marble system for the tissue regeneration therapy. The liquid marble technology was used with a paradigm-shifting concept. Rather than focus on the core, the technique was applied to develop a coating film, producing a multifunctional nanoparticulate shell. We first showed the possibility to produce hydrophobic BGNPs. The functionalized H-BGNPs exhibit no adverse effect on cell viability. Moreover, the obtained composites marbles – by combining H-BGNPs and gelatin – showed potential for the delivery of therapeutics since they could incorporate with high efficiency and sustain the release of two drug models: ibuprofen and albumin. The development of a bone-like layer on the exterior of the BHM upon immersion in SBF showed that the structures exhibited a bioactive behavior. The encapsulation of cells proved to be feasible, and viability studies demonstrated that the hydrogel core could sustain cell growth and proliferation. Moreover, the BHM could promote an early osteogenic commitment of encapsulated cells potentiating cell deliver therapies. Overall, the bio-inspired production method of BHM showed to be green, mild, reproducible, cost-effective, and highly efficient, originating a 3D hybrid system with high potential for bone tissue engineering applications. The BHM are envisioned to open new prospects in the development of bioactive shielded hydrogel systems to convey sensitive agents including not only cells and drugs but also genes and enzymes without adverse effects on their functionality. Also, a wise combination of core hydrogels with the attractive properties of ceramics (chemical, electrical, thermal, and mechanical), could bring up new possibilities and applications in distinct technological fields such as agrochemistry, biotechnology, cosmetics, and electronics, in where dynamic ceramic shells are often required.

## Methods

### H-BGNPs production and characterization

To produce the hydrophobic nanoparticles (H-BGNPs), we followed the methodology described in the Supplementary Information S1. Briefly, the chemical functionalization of BGNPs with 1 H,1 H,2 H,2 H-perfluorodecyltriethoxysilane (PFDTS, Sigma-Aldrich) originated the H-BGNPs^11^. The pristine and the modified nanoparticles were characterized for their morphology, chemical composition, size, wettability, and cytotoxicity. The samples were covered by a thin film of carbon through a sputter coater (EM ACE600, Leica, Germany) before analysis in a scanning electron microscope (SEM; JSM-6010LV, JEOL, Japan), operated at 15 kV to avoid damaging beam effects. X-ray photoelectron spectroscopy (XPS, Axis Supra, Kratos, UK) equipped with monochromatic Al (Kα) source operating at 1486.6 eV at 90° was used. The charge referencing was set at 285.0 eV of C1s hydrocarbon peak. Fourier transform infrared spectroscopy (FTIR, IRPrestige 21; Shimadzu, Japan) was performed in transmittance using KBr pellets (4000-400 cm^−1^, 32 scans, resolution of 2 cm^−1^). Dynamic light scattering (DLS, Malvern instrument 2000, UK) was performed at 25 °C, defining a minimum of 10 and a maximum of 100 runs, for 7 independent samples containing 1 mg mL^−1^ of nanoparticles dispersed in ethanol. Water and diiodomethane contact angles were measured in a nanoparticulate agglomerated plates using a goniometer (OCA 15 + , DataPhysics, USA), at room temperature, following the sessile drop method (5 μL per drop). The average values measured in five locations and the calculated surface energy (γ) were obtained using the SCA 20 software using the Owens, Wendt, Rabel, and Kaelble (OWRK) equation^64^. Human umbilical vein endothelial cells (HUVECs, ThermoScientific) and human osteosarcoma cells (SaOS-2, ATCC) were used as cell lines in cytocompatibility assessments that were performed on agglomerated plates. The labeling of nuclei and skeleton was achieved through a DAPI-phalloidin staining. For quantification of the cells number and area, all images were processed using specific algorithms developed in ImageJ (version 2.0, NIH, USA)^65^. The metabolic activity was analyzed by AlamarBlue assay (BUF012B, Arium). The medium was removed after each time point, and 1.5 mL of culture medium containing 10% (v/v) AlamarBlue was added. After incubation for 3 h at 37 °C, absorbance was measured using a plate reader (Biotek, synergy HT) at 570 and 600 nm. The percentage of AlamarBlue reduction was calculated according to the manufacturer’s recommendations. The data were normalized using the BGNPs condition at first time point as reference for each cell type line. See Supplementary Information S2 for details on the nanoparticles cytotoxicity screening.

### Bioactive hydrogel marble manufacture: A self-cleaning approach

Superhydrophobic platforms were produced as described elsewhere with subtle variations^66^. Then, a uniform layer of H-BGNPs was dispersed on the superhydrophobic platforms. As proof of concept, photocrosslinkable gelatin methacrylate was synthesized by adding methacrylate groups to the amine moieties of natural gelatin, according to a described methodology^67^. Then, droplets of gelatin methacrylate - 4% w/v in PBS solution with 0.25% w/v of Irgacure (Ciba Chemicals) - rolled over the superhydrophobic platform, until a complete saturation of H-BGNPs at the droplet surface, producing marbles with a dense and uniform layer. Afterwards, the obtained liquid marbles were cross-linked using UV light (365 nm, 11.4 W/cm^2^) for 30 s producing the BHM. Digital photographs of the marbles were obtained using a digital camera (Canon Inc., Tokyo, Japan). For fluorescent microscopy, the gelatin methacrylate was formerly stained with fluorescein and the H-BGNPs were stained with rhodamine. For visualization of the floating BHM, the gelatin methacrylate was dyed with methylene blue.

### *In vitro* bioactivity tests

Individual BHMs, with an approximate surface area of 15 mm^2^, were immersed in 30 mL of simulated body fluid (SBF) and incubated under orbital shaking conditions (30 rpm) for 1, 3, and 7 days at 37 °C. The preparation of SBF followed the well-established protocol described by Kokubo and Takadama^45^. Following the withdrawal of the samples from SBF, BHM were gently washed with distilled water, dehydrated, and dried at room temperature and then kept in desiccators until further characterization. The morphology and elemental composition of the BHM shell were assessed by SEM and energy dispersive spectroscopy (EDS, QUANTAX200 Bruker, Germany). The EDS profiles were obtained without any pre-coating. The mineralization of the BHM shell was also followed by micro-computed tomography (µ-CT, Scanco 20, Skyscan 1702, Belgium) operated at 95 keV. ImageJ was used to construct 3D images. The crystallinity patterns of the BHM shell after and before incubation in SBF were recorded on a Philips PW1700 Series diffractometer using Cu Ka radiation conducted at 40 kV and 40 mA (data were obtained between 5° and 80° 2*θ*). The calcium, phosphorous, and silicon concentrations in the SBF solution were measured by inductively coupled plasma optical emission spectroscopy (ICP OES; JY2000 2, Jobin Yvon, Horiba) versus standard solutions according to the manufacturer’s guidelines. At least, five samples were analyzed per condition and per time point.

### Drug delivery

Ibuprofen (IBU, MW 206.3 g mol^−1^) and bovine serum albumin (BSA, MW 66,000 g mol^−1^) were loaded into the BHM to investigate their ability to encapsulate and dual release low and high molecular weight molecules. Ibuprofen and BSA were first dissolved in PBS and then added to gelatin methacrylate (1 mg mL^−1^ and 100 µg mL^−1^, respectively). Five BHM were immersed in SBF (5 mL) under forced sink conditions and incubated at 37 °C (30 rpm). Half of the release medium was withdrawn at predetermined times and replaced by fresh SBF. IBU was quantified by UV-vis spectroscopy (Specord 40, Analytik Jena, Germany) at 265 nm, using calibrations curves generated by pre-defined concentrations. BSA was quantified by micro-BSA assay (Sigma-Aldrich) according to the manufacturer’s instructions.

### Cell encapsulation and induction of osteocommitment

A pre-osteoblast cell line, MC3T3-E1 (ATCC), was used to study the BHM applicability on the early osteodifferentiation. MC3T3-E1 cells were dispersed in gelatin methacrylate (1 × 10^6^ cells mL^−1^) and encapsulated in the BHM. The BHM stayed in cultured for 7 days, without osteogenic supplements. For each time point, at least 5 samples were used. See Supplementary Information S3 for details on MC3T3-E1 culture conditions. For the live*-*dead staining, the BHM were incubated with calcein-AM (2 mL, 2 μg mL^*−*1^, Life Technologies) and propidium iodide (2 mL, 1 μg mL^*−*1^, Life Technologies). The nuclei were stained with 4′,6*-*diamidino*-*2*-*phenylindole (DAPI, 1:1000, Life Technologies). F*-*actin was stained with phalloidin (1:1000, Sigma*-*Aldrich). A reflected fluorescence microscope (Zeiss, Germany) was used for image acquisition. The immunodetection of alkaline phosphatase (ALP) was performed using Novex® AP Chromogenic Substrate (Invitrogen, USA). The solution of 5*-*bromo*-*4*-*chloro*-*3*-*indolyl*-*1*-*phosphate (BCIP) and nitroblue tetrazolium (NBT) forms a black*-*purple insoluble precipitate upon reaction with ALP. Cells were fixed in 3.7% formaldehyde in PBS for 20 min and permeabilized for 4 min with Tris–buffered saline (TBS, 5 mL, 0.05 M Tris*-*HCL, 0.15 M NaCl, 0.1% Tween 20, pH 7.5, all from Sigma*-*Aldrich). Then, the BHM were briefly rinsed with pure water for 2 min, and the solution was decanted. Novex® Chromogenic Substrate was added (3 mL) to cover the BHM. The samples were incubated for 5 hours at 30 rpm and then were washed with pure water to stop the reaction and maintained in darkness. Optical micrographs were obtained using a stereomicroscope (Stemi 2000 C, Germany) equipped with a digital camera. Cross-sections of the BHM were obtained using parallel razors with a gap of approx. 2 mm.

### Statistical analysis

Each analysis was conducted with, at least, independent triplicates. First, a Shapiro-Wilk test was applied to determine the data normality. The results showed that non-parametric test should be used for all comparisons. Results are exhibited as the mean ± standard deviation. Statistical analysis was done by ANOVA with post hoc Tukey test, using GraphPad Prism v7.00 software (San Diego, USA). Statistical significance was established at a p-value lower than 0.0332.

## Electronic supplementary material

Video S1. Cleaning Trails

Video S2. Bioactive Marble: Self Assembly

Video S3. Bioactive Marble: Bioactivity

Supplementary Information

## Data Availability

The authors declare that the data supporting the findings of this study are provided in the article and its Supplementary Information.
