# Supplementary material for: Bioactive Hydrogel Marbles
Source: Sci Rep. 2018 Oct 12;8:15215. doi: 10.1038/s41598-018-33192-6 (PMC6185919; doi:10.1038/s41598-018-33192-6)
Supplement: Supplementary file 4 — Supplementary Information [file 41598_2018_33192_MOESM4_ESM.pdf]

Supplementary Information

Bioactive hydrogel marbles

Álvaro J. Leite[1, 2], Nuno M. Oliveira[1, 2], Wenlong Song[3], João F. Mano*[,1,2,4]

[1] *3B's Research Group – Biomaterials, Biodegradables and Biomimetics, University of Minho, Headquarters of the European Institute of Excellence of Tissue Engineering and Regenerative Medicine, Avepark – Parque de Ciência e Tecnologia, Zona Industrial da Gandra, 4805-017 Barco, Guimarães (Portugal)*

[2] *ICVS/3B's - PT Government Associate Laboratory, Braga/Guimarães, Portugal*

[3] *The State Key Lab of Supramolecular Structures and Materials. Jilin University. Changchun, 130023 P.R. China*

[4] *Present address: Department of Chemistry, CICECO, University of Aveiro, 3810-193 Aveiro, Portugal. E mail: jmano@ua.pt*

[*] *Corresponding Author*

Supplementary Information S1

The preparation of the ternary form of BGNPs, with the composition SiO_2:CaO:P_2O_5 (mol.%) = 55:40:5, followed a described sol-gel protocol comprising sequential reagent dissolutions that resulted in hydrolysis and polycondensation reactions.[1] Briefly, tetraethyl orthosilicate (TEOS, 99.90% pure, Sigma-Aldrich) was used as the silicon precursor, ammonium hydrogen phosphate (98%, maximum of 33% NH_3, Sigma-Aldrich) as the phosphorus precursor, calcium nitrate tetrahydrate (99%, Sigma-Aldrich) as the calcium precursor, citric acid monohydrate (99-100%, Sigma-Aldrich) to promote hydrolysis, ethanol, ammonium hydroxide solution (Sigma-Aldrich) as the jellifying agent and polyethylene glycol (PEG, Sigma-Aldrich) as a surfactant. The mixture of precursor solutions (7.639 g of calcium nitrate in 120 mL of distilled water, and 9.167 g of TEOS in 60 mL of ethanol/30 mL of citric acid 10% (w v^{-1})) was added drop-by-drop to an aqueous solution containing the phosphorus precursor (1.078 g of ammonium hydrogen phosphate in 1500 mL of distilled water). The pH was adjusted to 11.5 with ammonium hydroxide solution (Sigma-Aldrich). The obtained precipitate was stirred for 48 hours, followed by a 24 hour resting period. The

precipitate was washed three times with distilled water. An aqueous solution of PEG (200 mL, 2% (w v^{-1})) was added to the precipitate, followed by freeze-drying. Finally, the gel powder was calcined at 700 °C for 5 hours.

The chemical functionalization of BGNPs with 1H,1H,2H,2H-perfluorodecyltriethoxysilane (PFDTS, Sigma-Aldrich) originated hydrophobic nanoparticles (H−BGNPs).[2] First, the BGNPs were heated at 190 °C under vacuum for 2 h to promote water desorption and expose the surface silanol groups.[3] Then, the BGNPs (0.1 g) were dispersed in a PFDTS solution (1 mL at 1% v/v) prepared in ethanol, and the suspension was left to react at room temperature overnight. The solid phase was separated from the liquid by centrifugation and heated at 220 °C for 5 min to promote silane hydrolysis and condensation, and producing a stable fluorosilane layer on the BGNPs surface. Before use, the H−BGNPs were washed with ethanol to remove any unreacted PFDTS and dried at room temperature.

Supplementary Information S2

Human osteosarcoma cell line (SaOS-2, ATCC) cells were expanded in Dulbecco's Modified Essential Medium (DMEM, Invitrogen) with phenol red and supplemented with 10% (v/v) heat inactivated fetal bovine serum (FBS, ThermoScientific) and 1% (v/v) antibiotic-antimycotic solution (penicillin 100 units per mL and streptomycin 100 μg mL^{-1}; Gibco, UK). Human umbilical vein endothelial cells (HUVECs, ThermoScientific) were expanded in medium 199 (M199, Sigma-Aldrich) with phenol red and supplemented with 20% (v/v) heat-inactivated FBS (ThermoScientific) 1% (v/v) antibiotic-antimycotic solution (penicillin 100 units per mL and streptomycin 100 μg mL^{-1}; Gibco, UK), 1% (v/v) of Glutamax, 100 μg mL^{-1} of heparin and 50 μg mL^{-1} of endothelial cell growth supplement (ECGS, Invitrogen). Cells were grown in 150 cm^2 tissue culture flasks and incubated at 37 °C in a humidified air atmosphere of 5% CO_2. Every 3-4 days, fresh medium was added. At 90% of confluence, cells grown in tissue culture flasks were washed with sterile phosphate buffered saline (PBS) and subsequently detached with 0.05% trypsin-EDTA solution for 5 min at 37 °C in a humidified air atmosphere of 5% CO_2. Cell culture medium was added to inactivate the trypsin effect. The cells were then centrifuged at 1200 rpm at 25 °C for 5 min, and the medium was decanted. A cell suspension with a density of $1x10^6$ cells mL^{-1} was prepared. The agglomerated plates of BGNPs or H−BGNPs were fixed with double carbon tape into the bottom of the wells of anti-adherent 24-well plates to prevent floating. 50 μL of cell suspension were placed on top of the agglomerated plates of BGNPs or H−BGNPs and cells were left to adhere for 3 h in an incubator. Afterwards, each well was filled with 1 mL of

the cell culture medium and placed at 37 °C in 95% relative humidity and 5% CO_2 for 1, 3, and 7 days.

The labeling of the SaOS-2 and HUVECs was achieved through a DAPI-phalloidin staining. Before staining, the cells were rinsed with PBS followed by incubation with a DAPI-phalloidin solution (2 mL, 2 μg mL^{-1}, Life Technologies). Cells were then fixed for 5 min with a paraformaldehyde-based solution. Reflected fluorescence microscopy (Imager Z1m, Zeiss, Germany) was used for image acquisition. For quantification of cell number and cell area, all images were processed using algorithms developed in ImageJ (version 2.0, NIH, USA) and by using specific routines previously reported.[4, 5] Briefly, image stacks in the green channel were used as input. Images were then thresholded and segmented, and measured by features established in the software. Six images per condition were used.

Supplementary Information S3

MC3T3-E1 cells were expanded in a basal medium consisting of α-MEM (Gibco) with phenol red and supplemented with 10% (v/v) heat inactivated fetal bovine serum (FBS, ThermoScientific) and 1% (v/v) antibiotic-antimycotic solution (penicillin 100 units per mL and streptomycin 100 μg mL^{-1}; Gibco, UK). The cells grew in tissue culture flasks (150 cm^2) that where incubated at 37°C in a humidified air atmosphere of 5% CO_2. Every 3 days, fresh medium was added. The cells were then encapsulated in the BHM ($1x10^6$ cells mL^{-1} suspended in gelatin solution). Afterwards, the cell culture medium was exchanged by fresh α-MEM. Cells were cultured for up to 7 days. For each time point, at least 3 samples were used.

Table S1. Elemental composition of the studied nanoparticles from XPS analysis. The pristine BGNPs are mostly constituted by oxygen and silicon. The presence of carbon is likely due to surface contamination.[6]

Particles	C_{1s}	O_{1s}	Si_{2p}	F_{1s}
BGNPs	12.11±0.28	61.51±0.41	26.39±0.43	0.0±0.0
H-BGNPs	39.01±0.38	36.21±0.28	11.60±0.42	13.19±0.23

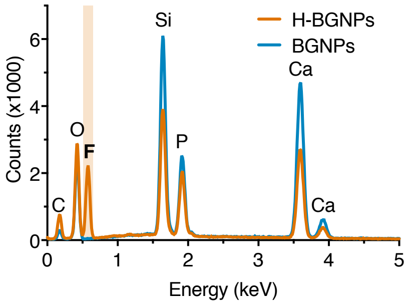

Figure S1. Identification of chemical elements using EDS.

Table S2. Calculated surface energy (γ) of the nanoparticles both after and before fluorosilanization. Water was used as polar liquid and diiodomethane as dispersive liquid. The surface energy was calculated using the Owens, Wendt, Rabel and Kaelble (OWRK) equation.[7]

**Surface**	**γd [mNm-^{1}]**	**γp [mNm-^{1}]**	**γ [mNm-^{1}]**
**BGNPs**	27.77±0.11	30.45±0.73	58.22±0.76
**H-BGNPs**	3.63±0.02	0.00±0.00	3.63±0.02

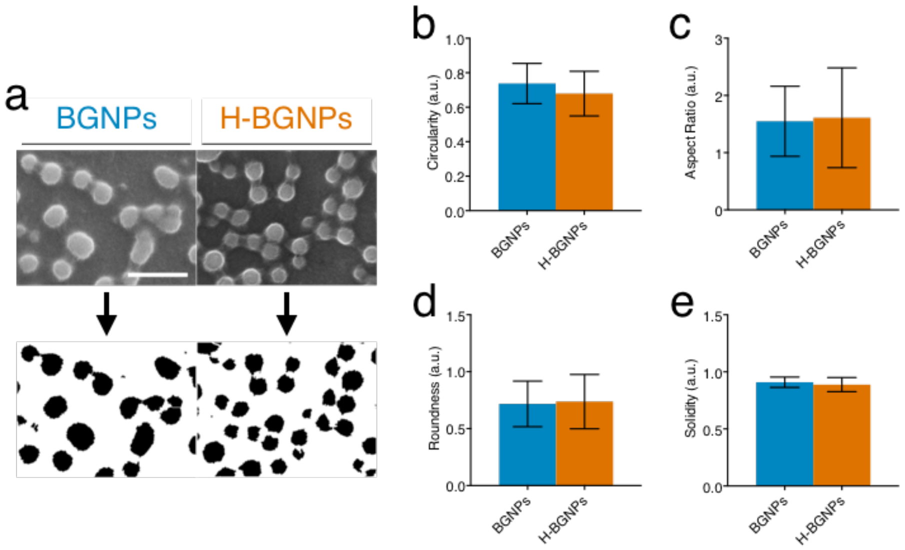

Figure S2. Shape descriptors analysis. **a)** Representative pictures of the image processing analysis by ImageJ. On top, the original SEM pictures, followed by the processed image on the bottom. Artifacts with an area higher than 10000 nm^2 were discharged as it likely represents agglomerates of nanoparticles. Images were analyzed in triplicates. Scale bar = 500 nm. **b)** Circularity $[4\pi \times (\text{Area} / \text{Perimeter})^2]$ with a value of 1.0 indicating a perfect circle and as the value approaches 0.0, it indicates an increasingly elongated shape. **c)** Aspect ratio of the particle, represented by [MajorAxis / MinorAxis]. **d)** Roundness, represented by $[4 \times (\text{Area} / \times \text{MajorAxis}^2)]$. **e)** Solidity, represented by [Area / ConvexArea].

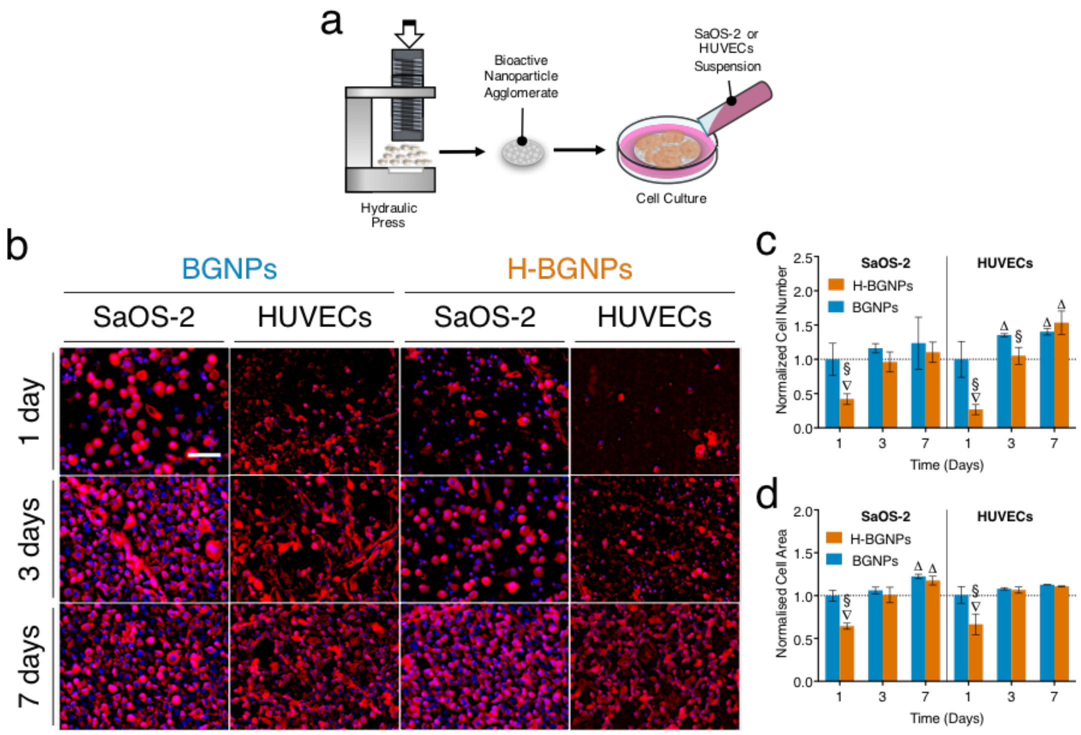

Figure S3. Biocompatibility assessment. **a)** Schematic representation of the in vitro cell biocompatibility studies. **b)** Representative photographs of calcein-AM staining of SaOS-2 and HUVECs during the cell culture time (scale bar = 200 μm). **c)** Normalized cell number of SaOS-2 and HUVECs cells seeded on BGNPs and H-BGNPs agglomerated samples based on an image analysis where each nucleus was detected as an object representing a cell. **d)** Normalized cell area of SaOS-2 and HUVECs cells seeded on BGNPs and H-BGNPs agglomerates. Cell area was determined using phalloidin signal analyzed after image processing to remove the background signal. These results showed that cells were evenly distributed over the surface of the agglomerated nanoparticles after 1, 3, and 7 days of culture. After 1 day of culture, the nanoparticles sustained cell attachment, though no statistical significance was found in cell proliferation from 3 to 7 days. These results also showed an absence of cytotoxicity of the nanoparticles, reinforcing their appropriateness in tissue engineering applications.

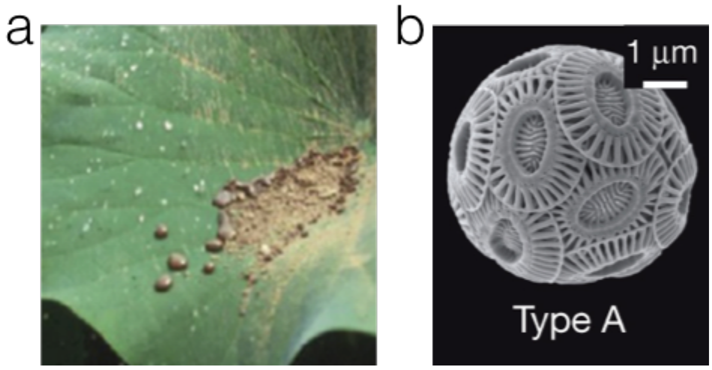

Figure S4. Inspiration by nature. **a)** The lotus effect or self-cleaning properties that result from hydrophobicity exhibited by the leaves of Nelumbo nucifera (lotus leaves). Adapted from [8]. Dirt particles are picked up by water droplets due to the micro- and nanoscopic architecture on the surface, which minimizes the droplet's adhesion to that surface. These surfaces with contact angles higher than 150°, exhibit extreme water repellency and have potential applications in the scientific and industrial fields.[9, 10] Self-cleaning properties are also found in other plants, on the wings of certain insects or when rain falls on hydrophobic soil created by a wildfire.[8, 11, 12, 13, 14] **b)** Coccolithophores (e.g. Emiliania huxleyi, adapted from [15]) are usually a spherical unicellular phytoplankton that lives in the upper layers of the world's oceans. These creatures can convert ions dissolved in seawater into calcium carbonate hard shells. [16, 17] The chemistry behind the process of shell-making, called marine calcification, relies on a complex series of chemical equations kept in a state of equilibrium, in a process analogous to bone formation.[18]

Table S3. Overview of the main signals detected in the FTIR spectra of BGNPs prior and after soaking in SBF for different time points.

Wavenumber [cm^{-1}]	Label	SBF Time	Description
1630	$\delta(H_2O)$	All Stages	Small Peak Coordinated water molecules adsorbed on the surface.[19]
1460	$\nu_3(CO_3^{2-})$	Before	Broad peak observed for symmetrically coordinated pure ionic carbonate ions adsorbed on surfaces.[20]
1420 & 1500	$\nu_3(CO_3^{2-})$	7 days	Bands can be attributed either to CO_3^{2-} adsorption or carbonate ions substituted in carbonated apatite.[21, 22, 23, 24]
1210	Si-O-Si	All stages	Formation of new Si-O-Si bonds on condensation of SiOH groups. Appearance of this band corresponds to the decrease of the SiO_{NBO} peak.[25, 26, 27]
1090	Si-O-Si	NA	Peak related to silicate network and ascribed to Si-O-Si asymmetric stretching.<br>Assigned to the asymmetric stretching mode Si-O-Si (s, asym).[24, 27]
1043	P-O	7 days	Phosphate absorption bands.[24]
1020	ν_{asym}(Si-O-Si)	Before	Broad complex band at 900-1200 cm^{-1} is difficult to analyze because of overlapping as both PO and SiO groups absorb in this region. Bending and stretching vibrations assigned to Si-O-Si, consistent with the silica network existing in the BGNPs.[24, 25, 28, 29]
963	P-O	7 days	Phosphate absorption bands.[24]
930-950	SiO_{NBO}	Before	Shoulder related to silicate network and ascribed to Si-O stretching of non-bridging oxygen atoms. The SiO_{NBO} is decreasing in intensity because of cation leaching.[24, 25, 27, 30]
871	$\nu_2(CO_3^{2-})$	Permanent	Band related to both carbonates adsorbed on the surface and CO_3^{2-} substituted in the apatite layer.[24, 28]
800	δ(Si-O-Si)	4 hours	Peak of 3D silica structures. Bending and stretching vibrations assigned to Si-O-Si, consistent with the silica network existing in the BGNPs. Assigned to the symmetric stretching vibration Si-O-Si(s, sym).[24, 25, 27, 29]
712	CO_3^{2-}	3 days	Small Peak characteristic band for calcite.[21]
570 & 601	P-O (cryst)<br>ν_4(P-O-P)	1-7 days	Double bonds at 601 and 568 cm^{-1} are the characteristic features of phosphate in crystalline phases.[24] Bending mode of P-O-P bond found in hydroxyapatite.[28]
560-610	P-O	4 hours	Attributed to the asymmetric vibration of $PO^{4}{}_{3-}$.[24, 25, 27]
490-500	ν_{sym}(Si-O-Si)	All stages	Bending and stretching vibrations assigned to Si-O-Si.[24, 25, 29, 30] Peak can shifted to 450 cm^{-1} because of leaching of cations.
470	P-O	7 days	Band phosphate absorption bands.[24]

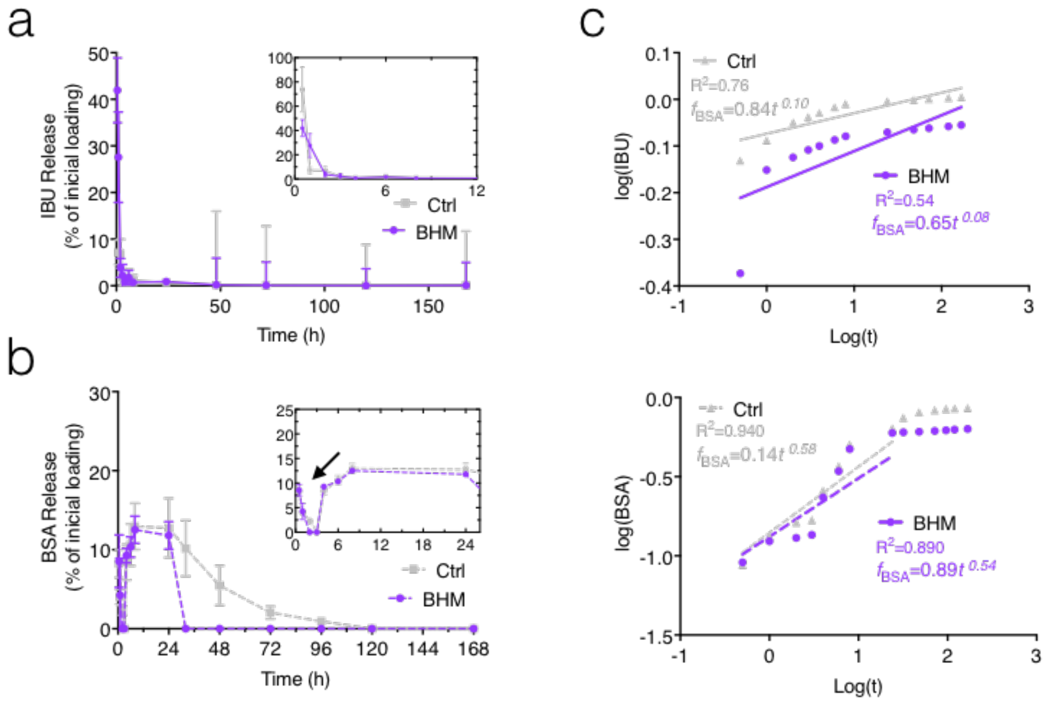

Figure S5. Release analysis and modeling of the release kinetics. **a)** IBU release profiles. The IBU release showed an abrupt release (burst effect), agreeing with other reports, as low molecular weight agents are more likely to have burst release profiles.[31] **b)** BSA release profiles. BSA had a slower release profile than IBU. The profile showed a small release at early times (arrow in inset) that we attributed to the rapid diffusion of the BSA molecules near to the sphere interface. Such release kinetics is consistent with the typical release of molecules having high molecular weight from hydrogel systems.[31] Compared with the control (hydrogels spheres without shell), the BSA released from BHM ceased the release between 1 and 3 days, indicating that the bioactive shell influenced the release. **c)** Modeling of the release kinetics by the power law described by Korsmeyer-Peppas. This model was developed to describe releases from polymeric matrices. The semi-empirical equation is given by Mi/M∞=ktn, where Mi is drug released over time t, M∞ is drug at the equilibrium state (≈ initial drug dosage), k is the release velocity constant, and n is the release exponent in function of time t (related to the release mechanism) and determined by the portion of release curve where Mi/M∞<0.60.[32] The calculated n values for IBU were <0.43 (theoretical value for spheres),[32] which implies a Fickian model, where diffusion controlled the release kinetics of IBU. The IBU transport rate or diffusion is higher than the process of polymeric chain relaxation. The calculated n values for BSA were between 0.43<n<0.85 (theoretical value for spheres),[32] suggesting a non-Fickian model, in where diffusion and swelling governed the release of BSA. The rearrangement of polymeric chains occurs slowly and the diffusion of BSA simultaneously causes a time-dependent release effect. Therefore, we hypothesize that the produced BHM can be used for a variable rate releases in a dual-drug administration strategy. The BHM could deliver a prime active agent at high release rates in the beginning of the treatment (e.g. providing immediate relief), followed by a prolonged release of a second active agent to promote gradual healing.

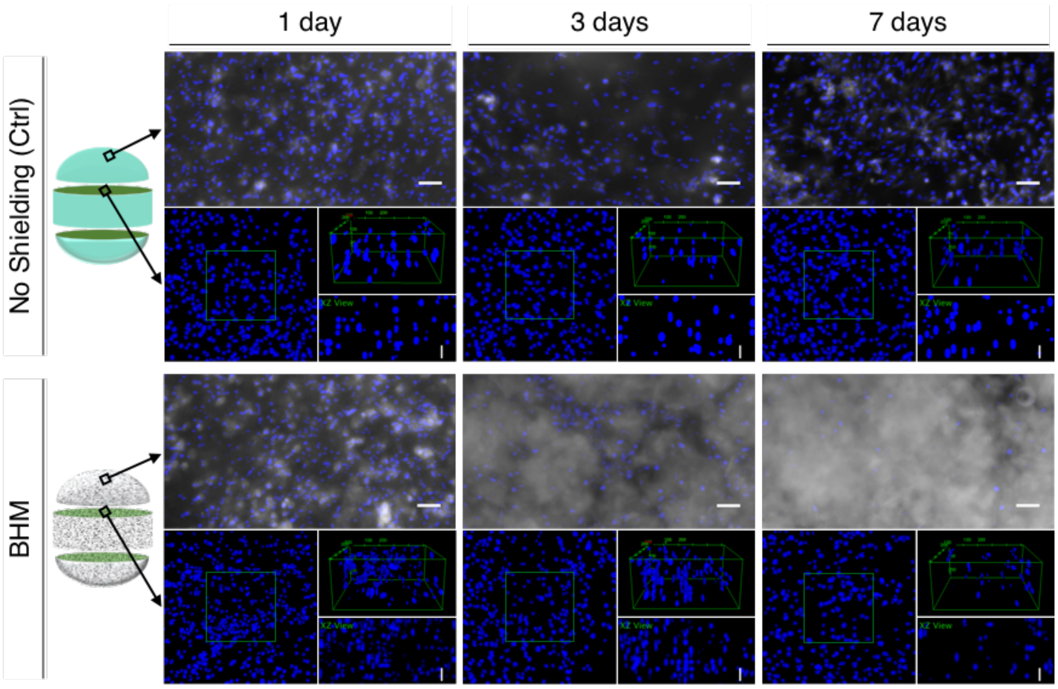

Figure S6. Surface and depth analyses of MC3T3-E1 cells inside the BHM. Each set presents a brightfield and DAPI image of the shell view, and DAPI image of core view with the region of interest, along with a 3D view, and a XZ view projection of the selected region. The calculation of the depth was based on the DAPI signal obtained by fluorescence microscopy. The likely position of the cells in-depth was obtained by the orthogonal image analysis of a stacks of z-slices. An area in the center of the marbles was choose as the region of interest.

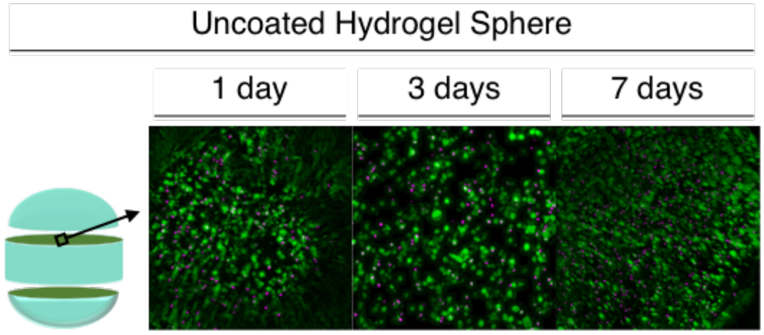

Figure S7. Live-dead fluorescent microscopy images (green represents live cells, and magenta represents dead cells) of MC3T3-E1 cells in non-coated hydrogels. Scale bar = 100 μm.

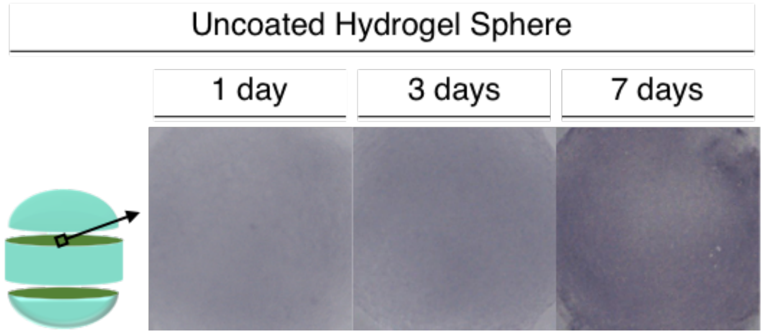

Figure S8. ALP images of MC3T3-E1 cells in non-coated hydrogels. Scale bar = 100 μm.

Table S4. Pearson correlation values.

	Time (days)	F Expression	Si Expression	P Expression	Ca Expression	Ca/P Expression	Ca Release	P Release	Si Release	Thickness	IBU Release	BSA Release	Cell Number	% Live Cells	Osteo. Commit.
**Time (days)**		-1.0	-1.0	0.9	0.9	0.9	-1.0	1.0	1.0	1.0	0.7	1.0	0.6	0.6	0.9
**F expression**	-1.0		1.0	-0.9	-0.8	-0.8	1.0	-1.0	-1.0	-1.0	-0.7	-1.0	-0.6	-0.6	-0.9
**Si Expression**	-1.0	1.0		-0.9	-0.8	-0.8	1.0	-1.0	-1.0	-1.0	-0.7	-1.0	-0.6	-0.6	-0.9
**P Expression**	0.9	-0.9	-0.9		1.0	1.0	-0.9	0.9	0.9	0.8	0.7	0.9	0.6	0.7	0.9
**Ca Expression**	0.9	-0.8	-0.8	1.0		1.0	-0.9	0.9	0.8	0.7	0.7	0.8	0.6	0.7	0.9
**Ca/P Expression**	0.9	-0.8	-0.8	1.0	1.0		-0.9	0.9	0.8	0.7	0.8	0.8	0.7	0.8	0.9
**Ca Release**	-1.0	1.0	1.0	-0.9	-0.9	-0.9		-1.0	-1.0	-0.9	-0.8	-1.0	-0.7	-0.8	-1.0
**P Release**	1.0	-1.0	-1.0	0.9	0.9	0.9	-1.0		1.0	1.0	0.8	1.0	0.6	0.7	0.9
**Si Release**	1.0	-1.0	-1.0	0.9	0.8	0.8	-1.0	1.0		1.0	0.8	1.0	0.6	0.7	0.9
**Thickness**	1.0	-1.0	-1.0	0.8	0.7	0.7	-0.9	1.0	1.0		0.7	1.0	0.5	0.6	0.8
**IBU Release**	0.7	-0.7	-0.7	0.7	0.7	0.8	-0.8	0.8	0.8	0.7		0.8	1.0	1.0	1.0
**BSA Release**	1.0	-1.0	-1.0	0.9	0.8	0.8	-1.0	1.0	1.0	1.0	0.8		0.6	0.7	0.9
**Cell Number**	0.6	-0.6	-0.6	0.6	0.6	0.7	-0.7	0.6	0.6	0.5	1.0	0.6		1.0	0.9
**% Live Cells**	0.6	-0.6	-0.6	0.7	0.7	0.8	-0.8	0.7	0.7	0.6	1.0	0.7	1.0		0.9
**Osteo. Commit.**	0.9	-0.9	-0.9	0.9	0.9	0.9	-1.0	0.9	0.9	0.8	1.0	0.9	0.9	0.9	

Table S5. Statistically significant P values.

	Time (days)	F Expression	Si Expression	P Expression	Ca Expression	Ca/P Expression	Ca Release	P Release	Si Release	Thickness	IBU Release	BSA Release	Cell Number	% Live Cells	Osteo. Commit.
**Time (days)**		*	*	ns	ns	ns	*	*	*	ns	ns	*	ns	ns	**
**F expression**	*		**	ns	ns	ns	*	*	*	*	ns	*	ns	ns	*
**Si Expression**	*	**		ns	ns	ns	*	*	*	*	ns	*	ns	ns	*
**P Expression**	ns	ns	ns		*	*	ns	ns	ns	ns	ns	ns	ns	ns	ns
**Ca Expression**	ns	ns	ns	*		*	ns	ns	ns	ns	ns	ns	ns	ns	ns
**Ca/P Expression**	ns	ns	ns	*	*		ns	ns	ns	ns	ns	ns	ns	ns	ns
**Ca Release**	*	*	*	ns	ns	ns		*	*	ns	ns	*	ns	ns	*
**P Release**	*	*	*	ns	ns	ns	*		*	ns	ns	*	ns	ns	**
**Si Release**	*	*	*	ns	ns	ns	*	*		*	ns	**	ns	ns	*
**Thickness**	ns	*	*	ns	ns	ns	ns	ns	*		ns	*	ns	ns	ns
**IBU Release**	ns	ns	ns	ns	ns	ns	ns	ns	ns	ns		ns	*	*	ns
**BSA Release**	*	*	*	ns	ns	ns	*	*	**	*	ns		ns	ns	*
**Cell Number**	ns	ns	ns	ns	ns	ns	ns	ns	ns	ns	*	ns		*	ns
**% Live Cells**	ns	ns	ns	ns	ns	ns	ns	ns	ns	ns	*	ns	*		ns
**Osteo. Commit.**	ns	ns	ns	ns	ns	ns	ns	ns	ns	ns	ns	ns	ns	ns	

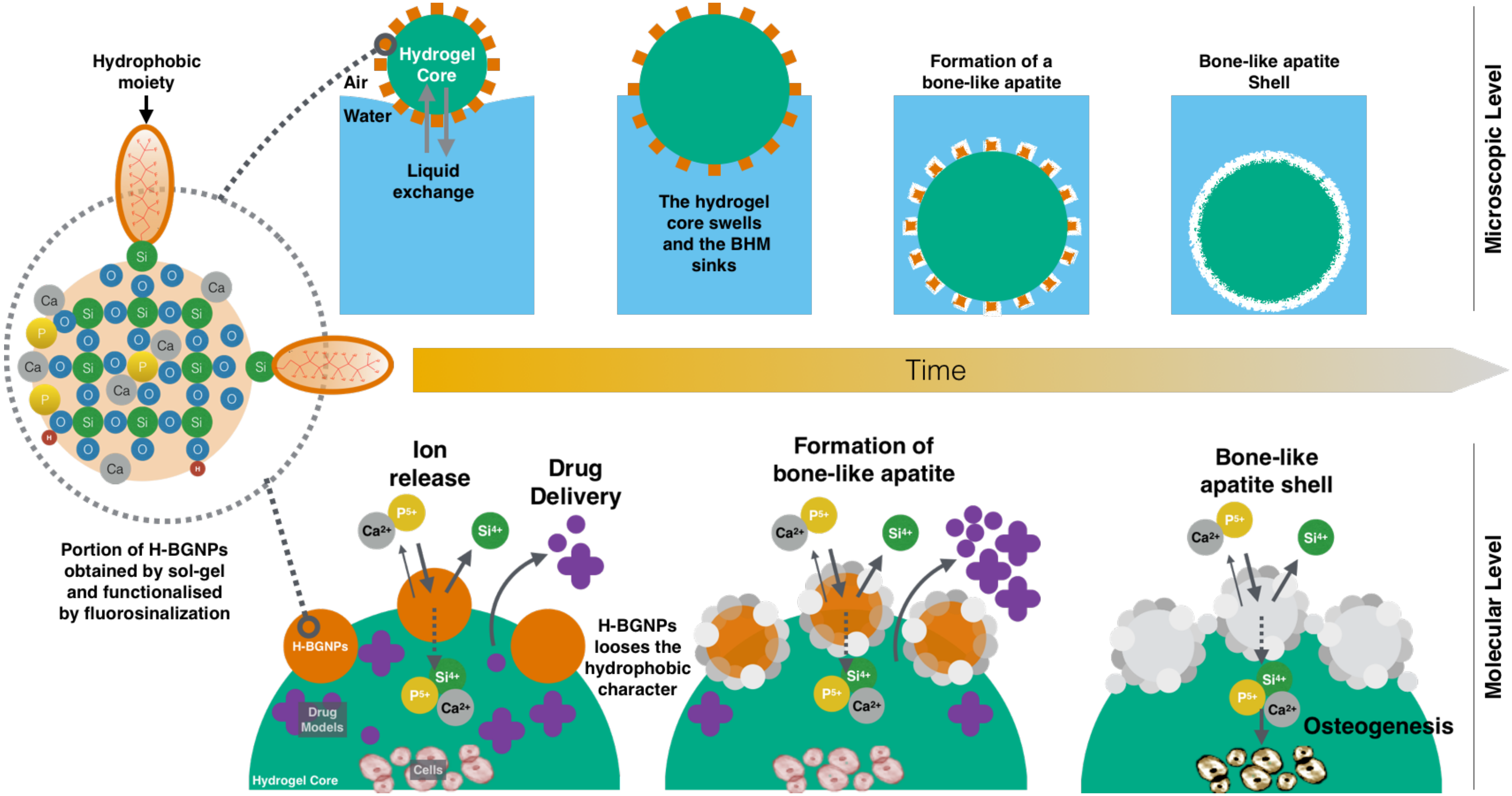

Figure S9. Scheme of the mechanism for the performance of the design BHM.

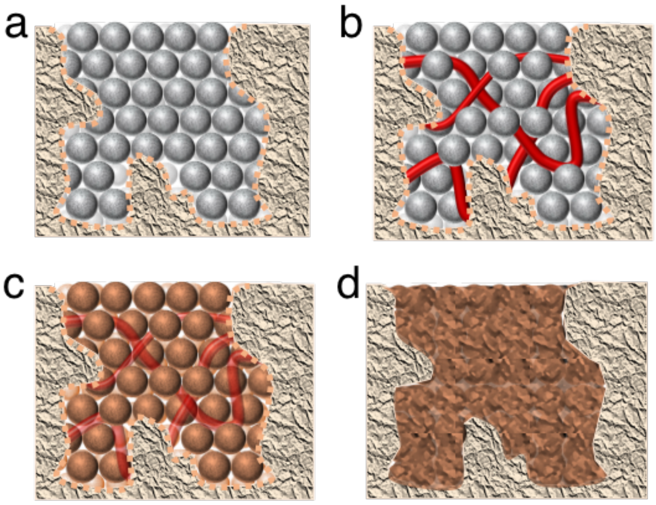

Figure S10. The predicted behavior of the produced bioactive hydrogel marbles in the regeneration of a bone defect. **a)** The ability of BHM to accommodate in intricate bone defects. When filling a space with spheres with approximately the same diameter, their tendency, due to their inherent symmetry, is to arrange themselves in a triangular order.[33, 34] This arrangement is the minimum energy configuration that leads to a stable state. Thus, the BHM could pack inside the defects by filling the geometrical void. **b)** Tissue infiltration and vascularization. The interstitial spaces between the spherical BHM may act as pores that allow oxygen and nutrient access and cell invasion. Consequently, the BHM can cumulate, ***in situ***, into organized 3D constructs with an interconnected porous structure. As we demonstrated the biocompatibility of H-BGNPs with osteosarcoma (SaOS-2) and endothelial cells (HUVECs), see **Figure S3**, we hypothesize that this feature can contribute to the healing process by bone tissue infiltration and vascularization, aiming the so-called angiogenic-osteogenic coupling. **c)** Upon implantation, the BHM could provide mineralization and deliver therapeutic drugs and cells. The BHM possesses a native high storage capacity as the spherical geometry has the smallest surface area per unit of volume. Comparing with non-spherical particles, for the same volume, they have a theoretical low drug flux per unit of volume which can contribute to a more sustained release.[35, 36] **d)** Osteointegration and new bone formation. The BHM could promote osteointegration by the formation of a bone-like apatite layer that can be remodeled by cells leading to an interfacial bonding with the bone.

References

1. Hong Z, Liu A, Chen L, Chen X, Jing X. Preparation of bioactive glass ceramic nanoparticles by combination of sol–gel and coprecipitation method. *Journal of Non-Crystalline Solids* **355**, 368-372 (2009).

2. Oliveira NM, Correia CR, Reis RL, Mano JF. Liquid Marbles for High-Throughput Biological Screening of Anchorage-Dependent Cells. *Advanced Healthcare Materials* **4**, 264-270 (2015).

3. Zhuravlev LT. The surface chemistry of amorphous silica. Zhuravlev model. *Colloids and Surfaces A: Physicochemical and Engineering Aspects* **173**, 1-38 (2000).

4. Schindelin J*, et al.* Fiji: an open-source platform for biological-image analysis. *Nat Meth* **9**, 676-682 (2012).

5. Schindelin J, Rueden CT, Hiner MC, Eliceiri KW. The ImageJ ecosystem: An open platform for biomedical image analysis. *Molecular Reproduction and Development* **82**, 518-529 (2015).

6. Yang Y, Jonas A-M, Dusan L. Functionalized diatom silica microparticles for removal of mercury ions. *Science and Technology of Advanced Materials* **13**, 015008 (2012).

7. Owens DK, Wendt RC. Estimation of the surface free energy of polymers. *Journal of Applied Polymer Science* **13**, 1741-1747 (1969).

8. Darmanin T, Guittard F. Superhydrophobic and superoleophobic properties in nature. *Materials Today* **18**, 273-285 (2015).

9. Song W, Veiga DD, Custódio CA, Mano JF. Bioinspired Degradable Substrates with Extreme Wettability Properties. *Advanced Materials* **21**, 1830-1834 (2009).

10. Jun S, Natália MA, João FM. Towards bioinspired superhydrophobic poly(L-lactic acid) surfaces using phase inversion-based methods. *Bioinspiration & Biomimetics* **3**, 034003 (2008).

11. Neinhuis C, Barthlott W. Characterization and Distribution of Water-repellent, Self-cleaning Plant Surfaces. *Annals of Botany* **79**, 667-677 (1997).

12. Koch K, Bhushan B, Barthlott W. Diversity of structure, morphology and wetting of plant surfaces. *Soft Matter* **4**, 1943-1963 (2008).

13. Cheng Y-T, Rodak DE. Is the lotus leaf superhydrophobic? *Applied Physics Letters* **86**, 144101 (2005).

14. Pike N, Richard D, Foster W, Mahadevan L. How aphids lose their marbles. *Proceedings of the Royal Society of London Series B: Biological Sciences* **269**, 1211-1215 (2002).

15. Read BA*, et al*. Pan genome of the phytoplankton Emiliania underpins its global distribution. *Nature* **499**, 209 (2013).

16. Weiner S, Dove PM. An Overview of Biomineralization Processes and the Problem of the Vital Effect. *Reviews in Mineralogy and Geochemistry* **54**, 1-29 (2003).

17. Iglesias-Rodriguez MD*, et al*. Phytoplankton Calcification in a High-CO₂ World. *Science* **320**, 336-340 (2008).

18. Luz GM, Mano JF. Biomimetic design of materials and biomaterials inspired by the structure of nacre. *Philosophical Transactions of the Royal Society A: Mathematical, Physical and Engineering Sciences* **367**, 1587-1605 (2009).

19. Cerruti M*, et al*. Surface Modifications of Bioglass Immersed in TRIS-Buffered Solution. A Multitechnical Spectroscopic Study. *The Journal of Physical Chemistry B* **109**, 14496-14505 (2005).

20. Cerruti M, Morterra C. Carbonate Formation on Bioactive Glasses. *Langmuir* **20**, 6382-6388 (2004).

21. Du H, Williams CT, Ebner AD, Ritter JA. In Situ FTIR Spectroscopic Analysis of Carbonate Transformations during Adsorption and Desorption of CO2 in K-Promoted HTlc. *Chemistry of Materials* **22**, 3519-3526 (2010).

22. Li P*, et al*. Process of formation of bone-like apatite layer on silica gel. *Journal of Materials Science: Materials in Medicine* **4**, 127-131 (1993).

23. Ohtsuki C, Kokubo T, Yamamuro T. Mechanism of apatite formation on CaOSiO2P2O5 glasses in a simulated body fluid. *Journal of Non-Crystalline Solids* **143**, 84-92 (1992).

24. Ma J, Chen C, Wang D, Meng X, Shi J. In vitro degradability and bioactivity of mesoporous CaO-MgO-P2O5-SiO2 glasses synthesized by sol–gel method. *Journal of Sol-Gel Science and Technology* **54**, 69-76 (2010).

25. Cerruti M, Greenspan D, Powers K. Effect of pH and ionic strength on the reactivity of Bioglass® 45S5. *Biomaterials* **26**, 1665-1674 (2005).

26. Jones JR, Sepulveda P, Hench LL. Dose-dependent behavior of bioactive glass dissolution. *Journal of Biomedical Materials Research* **58**, 720-726 (2001).

27. Wang H, Chen X, Wang Y, Cheng D. Preparation and characterization of the system SiO2-CaO-P2O5 bioactive glasses by microemulsion approach. *Journal of Wuhan University of Technology-Mater Sci Ed* **28**, 1053-1057 (2013).

28. Koutsopoulos S. Synthesis and characterization of hydroxyapatite crystals: A review study on the analytical methods. *Journal of Biomedical Materials Research* **62**, 600-612 (2002).

29. Correia CO, Leite AJ, Mano JF. Chitosan/bioactive glass nanoparticles scaffolds with shape memory properties. *Carbohydrate Polymers* **123**, 39-45 (2015).

30. Bunker BC, Tallant DR, Headley TJ, Turner GL, Kirkpatrick RJ. *The structure of leached sodium borosilicate glass*. Society of Glass Technology (1988).

31. Costa AMS, Alatorre-Meda M, Alvarez-Lorenzo C, Mano JF. Superhydrophobic Surfaces as a Tool for the Fabrication of Hierarchical Spherical Polymeric Carriers. *Small* **11**, 3648-3652 (2015).

32. Bruschi ML. Mathematical models of drug release. In: *Strategies to Modify the Drug Release from Pharmaceutical Systems* (ed^(eds). Woodhead Publishing (2015).

33. Barlow W. Probable Nature of the Internal Symmetry of Crystals. *Nature* **29**, 186 (1883).

34. Hales TC. An overview of the Kepler conjecture. *arXiv preprint math/9811071*, (1998).

35. Arifin DY, Lee LY, Wang CH. Mathematical modeling and simulation of drug release from microspheres: Implications to drug delivery systems. *Advanced Drug Delivery Reviews* **58**, 1274-1325 (2006).

36. Ritger PL, Peppas NA. A simple equation for description of solute release I. Fickian and non-fickian release from non-swellable devices in the form of slabs, spheres, cylinders or discs. *Journal of Controlled Release* **5**, 23-36 (1987).
